# Male Out-Migration: A Factor for the Spread of HIV Infection among Married Men and Women in Rural India

**DOI:** 10.1371/journal.pone.0043222

**Published:** 2012-09-06

**Authors:** Niranjan Saggurti, Bidhubhusan Mahapatra, Shrutika Sabarwal, Subash Ghosh, Aradhana Johri

**Affiliations:** 1 HIV and AIDS Program, Population Council, New Delhi, India; 2 National AIDS Control Organisation, Department of AIDS Control, Ministry of Health and Family Welfare, Government of India, New Delhi, India; National HIV and Retrovirology Laboratories, Canada

## Abstract

**Introduction:**

Thus far, the reasons for increasing HIV prevalence in northern and eastern Indian states are unknown. We investigated the role of male out-migration in the spread of human immunodeficiency virus (HIV) infection through a case-control study in rural India.

**Methods:**

Currently married men and women were recruited from HIV testing and treatment centers across seven selected districts with high rates of male out-migration in eastern and northern India in 2010 using a case-control study design. Case subjects (men: 595, women: 609) were people who tested HIV seropositive and control subjects (men: 611, women: 600) were those tested HIV seronegative. For each gender, we obtained adjusted odds ratios (AORs) and population attributable risks (PARs) for migration, and behavioral factors.

**Results:**

For men, the prevalence of HIV was significantly higher among those with a migration history (AOR, 4·4); for women, the prevalence of HIV was higher among those with migrant husbands (AOR, 2·3). For both genders, the returned male migration (men: AOR, 3·7; women: AOR, 2·8) was significantly associated with higher prevalence of HIV infection. The PAR associated with male migration was higher for men (54·5%–68·6%) than for women (32·7%–56·9%) across the study areas.

**Discussion:**

Male out-migration is the most important risk factor influencing the spread of HIV infection in rural areas with high out-migration rates, thereby emphasizing the need for interventions, particularly, for returned migrants and spouses of those migrants.

## Introduction

Migration plays a significant role in the spread of HIV/AIDS [1], [2]. Migration and mobility can increase vulnerability to HIV infection [3], [4], [5], [6] for mobile people [1], [7], [8], [9] and their partners residing at their hometown [10], [11]. The relationship between population migration and the spread of HIV in selected countries outside Asia is well documented [1], [4], [12]. For example, a study in Uganda found that individuals who had migrated in three years prior to survey were three times more likely to be infected with HIV than those who had not migrated during the past ten years prior to the survey [1]. Although migrant men are believed to acquire HIV infection in destination areas and transmit the virus to their sexual partners upon returning to their hometowns [13], [14], no epidemiological study has examined the relationship between spousal out-migration and the HIV serostatus of married women in rural India.

In India, barring a few micro-level studies conducted in urban destinations that have shown no differences in the sexual behaviors between migrants and non-migrants, [15], [16] most studies have documented a positive and significant correlation between migration and increased sexual risk behaviors [10], [17], [18], [19], [20], [21] and HIV [19]. A study of migrant and non-migrant youth in a district in rural Jharkhand, India indicates that migrants tend to have sexual relations with sex workers and casual female partners at their hometowns [20]. The likelihood of migrants engaging in sexual relations in their hometowns before their first migration and during regular home visits is higher than that of non-migrants [19], [20]. Although studies indicate high sexual risk behaviors in migrants' hometowns in India [22], there are no specific interventions aimed at migrants or their sexual partners in these settings due to lack of appropriate epidemiological data on the role of out-migration in the transmission of HIV to sexual partners in these areas. Further, recent evidence from India suggests that 85% of newly diagnosed HIV infections are among currently married persons [23]. Therefore, the current study is designed to examine this issue by comparing HIV seropositive and seronegative individuals by male out-migration status using a case-control study design. This study aimed to investigate whether a higher proportion of migrant married men—returned or active migrants—are HIV infected than non-migrants married men, and whether a higher proportion of married women—with returned or active migrant husbands—are identified with HIV infection than women with non-migrant husbands.

## Methods

### Study setting

The study was conducted in seven districts of India; three districts each in northern Bihar (Darbhanga, Muzaffarpur, and Sitamarhi) and eastern Uttar Pradesh (UP) (Azamgarh, Allahabad, and Deoria) and one district in Odisha (Ganjam). These seven districts are characterized by high male out-migration to other states because of lack of employment opportunities and urbanization (the rate of urbanization ranges from 6% in Sitamahri district to 25% in Allahabad district). Of a combined total population of 24.4 million in these seven districts, 5,11,286 people migrated to other states to seek employment by the year 2001 [24]. Of these, 4,83,027 were males who migrated to Delhi, Gujarat, Maharashtra, and West Bengal. The largest proportion of out-migrants from Ganjam migrated to Gujarat (mainly to Surat district) and Maharashtra (mainly to Mumbai/Thane districts), those from northern Bihar migrated to Delhi and West Bengal, and those from eastern UP migrated to Maharashtra. Coincidentally, HIV prevalence among female sex workers (FSWs) is high in metro cities that attract numerous rural male migrants from less developed states; HIV prevalence among female sex workers in Mumbai (Maharashtra), Surat (Gujarat), and Kolkata (West Bengal) are 37%, 8%, and 8%, respectively [25].

Results from a district level household survey conducted in 2007–08 suggest that these seven study districts are poorly developed as compared to national average: 20%–53% of households have electricity (all-India: 70%), 16%–27% have a toilet facility (all-India: 42%), and 66%–86% of households have low standard of living (all-India: 52%) [26]. In most districts, only a small percentage of women were aware that consistent condom use could reduce the risk of acquiring HIV/AIDS (21% in Ganjam to 45% in Allahabad), and a mere 1%–4% of the women had ever undergone HIV testing (all-India: 12%) [26]. Moreover, most households in these districts are socially disadvantaged; one-fourth of households belong to scheduled castes/tribes (a proxy for family's social status). The female illiteracy rate in these districts ranged between 36% to 67% (all-India: 35%) [27].

### Research design

A case-control study was conducted in 2010 wherein the cases were currently married HIV seropositive persons who had tested seropositive in the six months prior to the survey and were utilizing services from integrated counseling and testing centers (ICTCs) and/or antiretroviral therapy (ART) centers. Controls were currently married HIV seronegative persons who had been tested for HIV in the six months prior to the survey and were recruited from the same location as the cases. Respondent recruited in this case-control study were matched for their recruitment location in order to minimize the differences in HIV risk behaviors between cases and controls.

The sample size necessary for estimating migration's role in the spread of HIV infection was assumed for obtaining a result within a given percentage point of the true value with a 95% confidence interval (CI). It was assumed that, at most, 75% of the HIV-infected population would be migrants and the desirable precision of the estimation would be 5%, yielding a sample size of at least 200 cases and controls [28]. To achieve the target sample size within three months of data collection, three, four, and seven ICTCs in Ganjam, northern Bihar, and eastern UP respectively, and one ART center in each of the study districts were selected.

Research assistants contacted individuals who came to collect their HIV test results and/or receive counseling at the ICTCs to enquire about their willingness to participate in a study on HIV and health. Similarly, individuals who visited the ART centers (diagnosed for HIV at the ICTCs selected in the study) either for treatment pre-registration or further clinical investigations were contacted for their willingness to participate in the study. Interested individuals were requested to visit the interview venues located within campus of ICTCs and ART centers, where a research assistant assessed their eligibility for their study. The individuals' HIV serostatus was recorded in the survey instrument from the medical report card that was available with each individual who agreed to participate in the interview. The HIV test result was based on rapid enzyme linked immunosorbent assay (ELISA), a test to detect the presence of HIV antibodies; and the testing center confirmed positivity after conducting a second and third ELISA test. Interviewers fluent in English and local language of the state conducted the interviews with eligible respondents who consented to participate in the study. All the researchers had at least five years of relevant experience and a Graduate/Masters degree in sociology, anthropology, or statistics. A 45-minute interviewer-administered survey assessing the demographics, migration history of the respondent and spouse, sexual and condom use behaviors, risk factors for HIV infection, and sexually transmitted disease-related symptoms was answered by respondents in the local language of the state. Questionnaires were based on prior literature documenting HIV risk factors, including sexual behavior and migration in India [29], [30]. Instruments were developed in English, translated into local language of the state, and reviewed by investigators fluent in both languages. All interviews were conducted in private locations within the ICTCs and/or ART centers.

Overall, 1,300 women and 1,500 men from the seven districts were contacted. Of these, 1,250 women (HIV seropositive cases: 639; HIV seronegative controls: 611) and 1,230 men (HIV seropositive cases: 613; HIV seronegative controls: 617) met the study's eligibility criteria. Among those eligible, 41 women and 24 men refused to participate, or did not complete the interview and were thus excluded from the analyses, providing the final sample size of 1,209 women (HIV seropositive cases: 609; HIV seronegative controls: 600) and 1,206 men (HIV seropositive cases: 595; HIV seronegative controls: 611).

Procedures for this study were reviewed and approved by the institutional review boards of Population Council, and the ethics committee of the National AIDS Control Organization (NACO), Government of India. In accordance with the protocol, written informed consent was obtained from the participants prior to their participation.

Data quality control and questionnaire management involved immediate review by field staff after interviews to ensure accuracy and completion, same-day review by the field supervisor, and weekly transportation of survey forms to the data management team. To ensure consistency and accuracy, trained data entry officers entered and processed the survey data on a weekly and monthly basis respectively, through a customized data entry screen programmed using census and survey processing system (CSPro), a data entry software.

### Measures

Demographic data were single-item measures including age (>30 years, 18–29 years), education (no formal education, formal education), occupation (unemployed/unskilled work, skilled work), referral source (self/NGOs/CBOs/friends/relatives, hospital/health clinic), and residence (Ganjam, northern Bihar, or eastern UP).

Male and female out-migration status was assessed based on whether the respondents and/or their spouses had ever migrated for work. Further, respondents with a history of migration were asked whether they and/or their spouses were currently working in another state. Using these questions, two variables were constructed: Male/female out-migration, ever (yes, no) and Male/female out-migration status, current (never/returned/active migrant). As female out-migration was reportedly negligible, our analyses focused on two key independent variables: male out-migration ever and current status of male out-migration. For female respondents, spouse's out-migration was considered for analyses.

Men's sexual risk behaviors were assessed for the following types of sex partners: paid partners, unpaid casual partners, and male partners in case of the male survey. Respondents were asked to share their sexual experiences in destination and hometown areas, and were asked about their condom use behavior across sexual encounters. Coding details for these variables are provided as footnotes under each table.

The key outcome variable of interest in this study was the HIV serostatus of each respondent. Respondents were coded 1 and 0 if they were HIV seropositive and HIV seronegative, respectively.

### Statistical Analyses

Univariate and stratified multivariate analyses were conducted. A series of logistic regression models were constructed for married men and women samples to estimate the odds ratios (ORs) and 95% confidence intervals (CIs) for associations between male out-migration and HIV outcome. Adjusted population attributable risks (PAR) were also calculated for these variables using a procedure *aflogit* in STATA (version 10.0). Separate logistic regression models were used to estimate the ORs and 95% CIs for association between sexual risk behaviors and HIV outcomes within sub-samples where male out-migration status was non-migrant/returned migrant/active migrant. For these, a series of crude models and those adjusting for major demographics (age, education, occupation, residence, and referral source) were created.

## Results

### Socio-demographic characteristics of married men and women

A higher proportion of HIV seropositive married men than HIV seronegative married men had no formal education (33% vs. 24%, p<0·01), were unemployed or employed in unskilled occupations (87% vs. 79%, p<0·01), aged over 30 years (81% vs. 64%, p<0·01), and married for over 10 years (62% vs. 39%, p<0·01) (Table 1). Similarly, a higher proportion of HIV seropositive married women than HIV seronegative married women had no formal education (56% vs. 40%, p<0·01), were aged over 30 years (88% vs. 59%, p<0·01), and married for over 10 years (58% vs. 32%, p<0·01).

**Table 1:** Socio-demographic and sexual characteristics of HIV seropositive and HIV seronegative men and women—East and North India, 2010.

	Married men			Married women		
	HIV seropositive Men (n = 595) Number (%)	HIV seronegative Men (n = 611) Number (%)	AOR* ^,^ 1 (95% CI)	HIV seropositive Women (n = 609) Number (%)	HIV seronegative Women (n = 600) Number (%)	AOR* ^,^ 1 (95% CI)
Age above 30 years	481 (80·8)	389 (63·7)	1·5 (1·1–2·1)	535 (87·9)	357 (59·5)	3·3 (2·3–4·6)
No formal education	199 (33·4)	147 (24·1)	1·2 (0·9–1·6)	341 (56·0)	239 (39·8)	1·5 (1·1–1·9)
Unemployed or unskilled occupation	519 (87·2)	481 (78·7)	2·0 (1·4–2·8)	596 (97·9)	589 (98·2)	0·9 (0·4–2·1)
Referral: self/friends/NGOs	222 (37·3)	226 (36·9)	1·0 (0·8–1·4)	290 (47·6)	166 (27·7)	2·9 (2·1–3·8)
Duration of marriage >10 years	368 (61·9)	236 (38·6)	1·9 (1·4–2·6)	352 (57·8)	193 (32·2)	1·6 (1·2–2·1)
Reported > = 1 casual partners, lifetime	333 (55·9)	145 (23·7)	4·0 (3·1–5·2)	72 (11·8)	67 (11·2)	1·2 (0·8–1·8)
Reported sex with partner in exchange for money/gifts, lifetime	245 (41·2)	86 (14·1)	4·3 (3·2–58)	14 (2·3)	25 (4·2)	0·6 (0·3–1·1)

* AOR: adjusted odds ratio; model controlled for age, education, occupation, duration of marriage, type of referral, study district, and reported sex with partner in exchange of money/gifts at least once in their lifetime.

1 Dependent variable: HIV seropositive (yes/no).

### Association between male out-migration and own HIV serostatus

Among men, 80% of HIV seropositive respondents versus only 44% of the HIV seronegative respondents had ever migrated (Table 2). The multiple logistic regression analyses adjusted for socio-demographic characteristics demonstrate a significant association between migration and men' HIV status. The odds of HIV seropositivity were four times higher among men with a migration history than those who had never migrated (adjusted odds ratio (AOR) = 4·4, 95% CI = 3·3–5·9, p<0·01). The proportion of HIV-infection among married men in the study area that could be attributed to male out-migration history was estimated at 62% (95% CI = 54·5–68·6), of which, active migrant men's contribution was higher than that of returned migrant men.

**Table 2:** Male out-migration and HIV in East and North India, 2010.

	Married Men				Married Women			
	HIV seropositive Men (n = 595) Number (%)	HIV seronegative Men (n = 611) Number (%)	AOR* (95% CI)	PAR† (95% CI)	HIV seropositive Women (n = 609) Number (%)	HIV seronegative Women (n = 600) Number (%)	AOR* (95% CI)	PAR† (95% CI)
**Male out-migration history, ever**	478 (80·3)	271 (44·4)	4·4 (3·3–5·9)	62·2 (54·5–68·6)	501 (82·3)	388 (64.7)	2·3 (1·7–3·0)	46·1 (32·7–56·9)
**Male out-migration status, current^#^**								
Non-migrants	117 (19·7)	340 (55·6)	Referent	—	108 (17·7)	212 (35.3)	Referent	—
Returned migrants	139 (23·4)	75 (12·3)	3·7(2·3–5·8)	17·0 (12·3–21·4)	180 (29·6)	111 (18.5)	2·8 (1·9–4·3)	23·6 (17·4–28·7)
Active migrants	339 (56·9)	196 (32·1)	4·8 (3·5–6·7)	45·1 (39·0–50·6)	321 (52·7)	277 (46.2)	2·1 (1·5–2·9)	22·5 (15·3–28·1)
**Female out-migration for work, ever**	9 (1·5)	12 (2·0)	0·6 (0·2–1·5)	NE	9 (1·5)	6 (1.0)	1·5 (0·5–4·5)	0·5 (0·0–2·9)

* AOR: adjusted odds ratio; model controlled for age, education, occupation, duration of marriage, type of referral, study district, and reported sex with partner in exchange of money/gifts, lifetime.

† PAR: Population-attributable risk; CI: Confidence interval; NE: Not estimable.

### Association between spousal out-migration and women's HIV serostatus

Data from women survey revealed higher odds of HIV seropositivity among those whose husbands had a history of migration than those whose husbands had never migrated (82% vs. 65%, AOR = 2·3, 95% CI = 1·7–3·0, p<0·01) (Table 2). Compared to women whose husband had never migrated, the odds of HIV seropositivity were approximately three times higher for women whose husband was returned migrant (29% vs. 18%, AOR = 2·8, 95% CI = 1·9–4.3, p<0·01) and two times higher for females whose husband was active migrant (53% vs. 46%, AOR = 2·1, 95% CI = 1·5–2·9, p<0·01). Among married women, the proportion of HIV infection in the study areas that could be attributed to husband's out-migration history was estimated at 46% (95% CI = 32·7–56·9), of which, the contribution returned migrant men was similar to that of active migrant men.

### Male out-migration, sexual risk behaviors, and their association with married men's HIV serostatus

An analysis of sex risk behaviors by male out-migration status was conducted based on the survey data for married men (Table 3). Among active migrants, the multivariate models demonstrate that the odds of HIV seropositivity were higher if they reported having sex with a partner in exchange for money/gifts at least once in their lifetime (45% vs. 19%; AOR = 3·6, 95% CI = 2·3–5·7), having sex in migrant destinations (40% vs. 18%; AOR = 3·8, 95% CI = 2·3–6·2), or inconsistent condom use in sexual encounters along migration routes (94% vs. 74%; AOR = 5·4, 95% CI = 2·1–13·9) compared to their counterparts in these categories. Similar results were noted among returned migrant men. Returned migrant men who reported having sex with male partners (30% vs. 4%; AOR = 7·8, 95% CI = 1·9–33·3) or having extramarital sex in their hometown (32% vs. 4%; AOR = 6·3, 95% CI = 1·6–23·9) had higher odds of HIV infection than their counterparts. The odds of HIV seropositivity among non-migrants were higher if they reported having sex with at least one casual partner in their lifetime (32% vs. 20%, AOR = 2·0, 95% CI = 1·2–3·3), having sex with a partner in exchange for money/gifts at least once in their life time (21% vs. 11%, AOR = 2·1, 95% CI = 1·2–3·7), and/or having sex with a male partner (12% vs. 4%, AOR = 3·6, 95% CI = 1·6–8·2).

**Table 3:** Sexual/behavioral characteristics of HIV seropositive and HIV seronegative men by male out-migration status—East and North India, 2010.

	HIV seropositive Men (n = 595) Number (%)	HIV seronegative Men (n = 611) Number (%)	AOR* (95% CI)	PAR† (95% CI)
**Male: Active Migrant (n = 535)**	339	196		
Reported > = 1 casual partners, lifetime	203 (59·9)	62 (31·6)	3·0 (2·0–4·5)	40·1 (29·2–49·3)
Reported sex with partner with in exchange for money/gifts, lifetime	151 (44·5)	37 (18·9)	3·6 (2·3–5·7)	32·3 (24·1–39·6)
Reported sex with a male partner	34 (10·0)	10 (5·1)	1·8 (0·8–4·0)	4·6 (0·0–9·7)
Reported extramarital sex in city (destination)	137 (40·4)	35 (17·9)	3·8 (2·3–6·2)	29·7 (22·1–36·6)
Reported extramarital sex in native place either during migration or current visit (origin)	15 (4·4)	6 (3·0)	1·1 (0·4–3·2)	0·5 (0·0–5·0)
Inconsistent condom use in sexual encounters (N = 219)	153 (94·4)	42 (73·7)	5·4 (2·1–13·9)	76·9 (47·9–89·7)
**Male: Returned Migrant (n = 214)**	139	75		
Reported > = 1 casual partners, lifetime	93 (66·9)	14 (18·7)	5·4 (2·5–11·7)	54·5 (39·6–65·8)
Reported partner with exchange of money/gifts, lifetime	70 (50·4)	11 (14·7)	3·4 (1·5–7·8)	35·5 (18·3–49·0)
Reported sex with a male partner	42 (30·2)	3 (4·0)	7·8 (1·9–33·3)	26·4 (17·4–34·4)
Reported extramarital sex in city (destination)	93 (66·9)	15 (20·0)	7·4 (3·4–16·2)	57·9 (45·7–67·3)
Reported extramarital sex in native place either during migration or current visit (origin)	45 (32·4)	3 (4·0)	6·3 (1·6–23·9)	27·2 (16·9–36·3)
Inconsistent condom use along the migration route (N = 123)	91 (96·8)	23 (79·3)	7·0 (1·3–37·8)	82·9 (23·7–96·2)
**Male: Non-migrant (n = 457)**	117	340		
Reported > = 1 casual partners, lifetime	37 (31·6)	69 (20·3)	2·0 (1·2–3·3)	15·9 (3·5–26·6)
Reported sex with partner in exchange for money/gifts, lifetime	24 (20·5)	38 (11·2)	2·1 (1·2–3·7)	10·7 (1·0–19·4)
Reported sex with partner in exchange for money/gifts, 12 months	15 (12·8)	31 (9·1)	1·5 (0·7–2·9)	4·1 (0·0–11·7)
Reported sex with a male partner	14 (11·9)	14 (4·1)	3·6 (1·6–8·2)	8·7 (1·8–15·0)
Not used condom with male/female partner, last time (N = 116)	39 (90·7)	60 (82·2)	3·5 (0·9–12·9)	64·5 (0·0–88·3)

* AOR: adjusted odds ratio; model controlled for age, education, occupation, duration of marriage, type of referral, and study district.

† PAR: Population-attributable risk; CI: Confidence interval.

## Discussion

The increasing prevalence of HIV in districts with high male out-migration in India [31] has raised important questions regarding the role of migration in the spread of HIV. This study indicates that the proportion of migrant men among HIV seropositive individuals is higher than that among HIV seronegative individuals in rural India, suggesting a concentration of HIV among individuals with migration history. These findings are consistent with the findings of previous studies conducted among African and Asian populations [10], [32], [33].

For men, sexual risk behaviors are strongly associated with HIV seropositivity. The relationship between sexual risk behaviors and HIV seropositivity are much stronger among the group of migrant men than non-migrant men. The high odds of HIV seropositivity among active migrants reporting extramarital sex in destination areas suggest that they may have been infected with HIV in those areas. Additionally, a higher proportion of returned migrants reporting extramarital sexual relationships in hometowns suggest that they may be contributing significantly to the spread of HIV in their hometowns; these findings are consistent with the some of the study results from Nepal [33] and India [22]. The lesser odds of being HIV seropositive among non-migrant than migrant men reporting extramarital sexual relationships indicates the high concentration of the epidemic among migrant families and lower HIV prevalence among men from non-migrant families. These are important findings given the widespread prevalence of the HIV epidemic in western Indian states among various high risk population groups (such as female sex workers, men who have sex with men), which attract numerous migrants from other parts of the country. It is likely that migration plays an important role in the spread of HIV from high HIV prevalence states of India to migrants' hometowns.

Although the overall HIV seropositivity rates are lower among women than men, the results indicate the occurrence of HIV infection among married women from their HIV seropositive husbands in rural areas. Further, the results suggest that being a partner of either returned or active migrant presents significant level of risk than being a partner of non-migrant men. In addition to HIV risk from migrant husbands, one could argue that women may also be migrating for work and the environments around such migration may be putting them at risk too. The data in the current study indicates that less than 2% of the women reported a history of migration for work from these areas, making it difficult to examine the association between women's migration and HIV infection. Nevertheless, the weak association between female migration and HIV seropositive status raises important research questions regarding the extent to which HIV risk among women could be attributed to: their own extramarital sexual behaviors, lack of knowledge about modes of HIV transmission and prevention, and lack of condom use in marital sex. Further in-depth studies are needed to examine these issues.

One of the strength of this study is that almost all HIV seropositive married men and women (cases) were recruited from HIV testing and treatment centers in high out-migration areas and controls were randomly selected from the same centers; therefore, the findings should accurately reflect migration's role in the spread of HIV infection. More importantly, both cases and controls were newly diagnosed HIV seropositive and HIV seronegative individuals, indicating some causal relationship between history of migration and HIV. The non-response rates among cases and controls were low, thereby reducing the likelihood of selection bias in the explanation of our key findings.

An unavoidable study limitation is that the relationship between active migrant men and HIV status may not be indicative of an actual relationship, as it included only a selective sample of men who were present in the hometown (study location) at the time of study. While under-representation of active migrant men should not compromise the internal validity of the study; rather it may have resulted in an underestimation of the population's risk of being infected with HIV that could be attributed to migrant men's behavior. Another limitation is related to study focus being assessing the role of male out-migration in the spread of HIV among women; other determinants of HIV transmission among women at the population level, such as their own extramarital sexual behavior, have not been captured. Further, most individuals who were tested for HIV at ICTCs are volunteers or referred by NGOs/friends whose levels of HIV risk behaviors may differ from those in the general population. Although this may limit the generalization of the study findings, one can argue that if individuals are recruited from general population using a case-control study design matched for HIV seropositive and HIV seonegative, the ratio of migrants to non-migrants among HIV seropositive and seronegative individuals may remain same as observed in this study. Although future population based research could address some of these limitations, this large-scale case-control study offers, for the first time, empirical evidence on the role of male out-migration in the spread of HIV in districts with high male out-migration.

Overall, this study shows that migrant men and their partners are at higher risk of HIV infection than their non-migrant counterparts, thereby confirming the concentration of the epidemic among migrants and their partners in destination and hometown areas and underscoring the importance of designing HIV prevention programs for these groups in both settings. The high volume of returned migrants and persistently high HIV prevalence among migrants and their spouses in their hometown reflect the urgent need to provide HIV prevention and treatment services in these areas. However, targeting only migrants and their spouses in rural areas can increase stigma and discrimination; therefore, integrating HIV prevention and treatment services with existing structural resources within rural settings, such as public health centers, village administrative (*panchayat*) offices, and HIV testing and treatment centers may be more effective. Appropriate operations research is required to test the feasibility and effectiveness of such structural interventions in India and elsewhere.
